# A novel extension of half-logistic distribution with statistical inference, estimation and applications

**DOI:** 10.1038/s41598-024-53768-9

**Published:** 2024-02-21

**Authors:** A. A. Bhat, S. P. Ahmad, Ahmed M. Gemeay, Abdisalam Hassan Muse, M. E. Bakr, Oluwafemi Samson Balogun

**Affiliations:** 1 Department of Mathematical Sciences, Islamic University of Science and Technology, Awantipora, 192122 India; 2 Department of Statistics, University of Kashmir, Srinagar, 19006 India; 3 Department of Mathematics, Faculty of Science, Tanta University, Tanta, 31527 Egypt; 4 Faculty of Science and Humanities, School of Postgraduate Studies and Research (SPGSR), Amoud University, Borama, 25263 Somalia; 5 Department of Statistics and Operations Research, College of Science, King Saud University, P.O. Box 2455, 11451 Riyadh, Saudi Arabia; 6 Department of Computing, University of Eastern Finland, 70211 Joensuu, Finland

**Keywords:** Odd Frechet-G family, Half logistic distribution, Quantile function, Statistical properties, Monte Carlo simulation, Estimation methods, Computational science, Statistics

## Abstract

In the present study, we develop and investigate the odd Frechet Half-Logistic (OFHL) distribution that was developed by incorporating the half-logistic and odd Frechet-G family. The OFHL model has very adaptable probability functions: decreasing, increasing, bathtub and inverted U shapes are shown for the hazard rate functions, illustrating the model’s capacity for flexibility. A comprehensive account of the mathematical and statistical properties of the proposed model is presented. In estimation viewpoint, six distinct estimation methodologies are used to estimate the unknown parameters of the OFHL model. Furthermore, an extensive Monte Carlo simulation analysis is used to evaluate the effectiveness of these estimators. Finally, two applications to real data are used to demonstrate the versatility of the suggested method, and the comparison is made with the half-logistic and some of its well-known extensions. The actual implementation shows that the suggested model performs better than competing models.

## Introduction

In the field of probability distribution theory, the normal and exponential distributions serve as fundamental models, illustrating various theoretical findings. For instance, the exponential distribution is often employed as a constant example, particularly in reliability research. However, the use of exponential distribution is incompatible in conditions wherein failure rate is not constant. To integrate this situation, extensions like the Weibull and gamma distributions are used, offering solutions to model non-constant failure rates. The Weibull and gamma distributions can be seen as expansions of the exponential distribution, providing more flexibility in handling varying failure rates. Additionally, the half logistic model, resembling the shape and characteristics of the Weibull distribution, serves as another alternative in such cases, offering a different approach to modeling scenarios with non-constant failure rates.

The half-logistic (HL) model introduced by Balakrishnan^1^ using the absolute transformation of the logistic distribution is highly regarded in modelling datasets from a wide range of fields and has got tremendous importance in statistics, physics, hydrology and logistic regression. The best unbiased estimators of the parameters of the HL model were discovered by Balakrishnan and Puthenpura^2^ using linear functions of order statistics. Balakrishnan and Wong^3^ utilised type II censoring in order to determine the approximate maximum likelihood estimates of the HL model. Some characterizations results in the form of theorems of the HL model were studied by Olapade^4^. The reliability test plan for the HL distribution developed by Rosaiah et al.^5^ among others are some important sources of the HL distribution.

The probability density function (PDF) and cumulative distribution function (CDF) of Half-Logistic (HL) model with shape parameter $\theta$ are

1 $$m\left(x;\theta \right)=\frac{2\theta {e}^{-\theta x}}{{\left(1+{e}^{-\theta x}\right)}^{2}} ; x,\theta >0$$

and

2 $$M\left(x;\theta \right)=\frac{1-{e}^{-\theta x}}{1+{e}^{-\theta x}} ; x,\theta >0$$

In practice, lifetime studies call for more adaptable models that can take into account various types of data sets. In light of these issues, several authors have focused on the HL model in recent years, offering a range of extensions and novel forms. For example, the generalised half-logistic model with extended properties was reported by Torabi and Bagheri^6^, who also investigated several estimation methodologies for estimating the parameters of the model using both complete and censored data. Rao et al.^7^ made an effort to present the basic characteristics, parameter estimation and hypothesis testing of the exponential half-logistic distribution. Olapade^8^ proposed the Odd generalized half-logistic model. Cordeiro et al.^9^ investigated the exponentiated half-logistic family of distributions to study the extension of the HL model. Krishnarani^10^ studied the power transformation of HL model and derived its different characterization results while as Yegen and Özel^11^ extended the half-logistic distribution by utilizing Marshall-Olkin-G approach.

In statistical theory, generalizing probability distributions is a common practice. To generate adaptable mathematical models that can handle non-normal data circumstances, new distributions are being developed. This flexibility can be obtained in a straightforward manner by including additional parameters such as location, scale and shape. There are several generalized (or G) classes that have recently emerged in the literature, including the Weibull-G family proposed by Bourguignon et al.^12^, Weighted Weibull-G family of distributions by Hassan et al.^13^, Kumaraswamy-G family of distributions developed Cordeiro and de Castro^14^, two innovative methods for generating probability models with application to Weibull distribution were proposed by Lone et al.^15,16^.

Recently, an intriguing technique “Odd Frechet-G (OF-G)” family of probability distributions was presented by Haq and Elgarhy^17^ with CDF and PDF respectively given as

3 $$F\left(x;\alpha ,\pi \right)=\underset{0}{\overset{\left[\frac{M(x;\pi )}{1-M(x;\pi )}\right]}{\int }}\frac{\alpha }{{x}^{\alpha +1}}{e}^{-{x}^{-\alpha }}dx=exp\left[-{\left(\frac{1-M\left(x;\pi \right)}{M\left(x;\pi \right)}\right)}^{\alpha }\right]$$

and

4 $$f\left(x;\alpha ,\pi \right)=\frac{\alpha m\left(x;\pi \right){\left[1-M\left(x;\pi \right)\right]}^{\alpha -1}}{{M\left(x;\pi \right)}^{\alpha +1}}exp\left[-{\left(\frac{1-M\left(x;\pi \right)}{M\left(x;\pi \right)}\right)}^{\alpha }\right]$$

where $m\left(x;\pi \right)$ is the PDF, $\pi$ is the parameter vector and $\alpha$ is the shape parameter of the baseline model.

The OF-G family is used to broaden the scope of the baseline distribution applicability in modeling different types of datasets. Utilising the OF-G approach, ZeinEldin et al.^18^ offered a novel generalisation of the inverse Lomax model, Elgarhy and Alrajhi^19^ investigated the distributional characteristics and applications of odd Frechet Inverse Rayleigh model, odd Frechet inverse exponential model was studied by Alrajhi^20^, odd Frechet inverse weibull model using OF-G scheme was suggested by Fayomi^21^ and Ahsan ul Haq et al.^22^ proposed odd Frechet power function distribution.

This study aims to introduce a novel distribution, the Odd Frechet Half Logistic (OFHL) model, by incorporating the Half Logistic (HL) distribution into the larger context of the OF-G family of distributions. The rationale behind adopting the OF-G family of distributions lies in its capacity to enhance the flexibility of the HL distribution. Moreover, utilizing the OF-G framework enables us to delve into the tail properties of the distribution and enhance its goodness-of-fit characteristics, thus offering a more comprehensive understanding and application of the OFHL model.

The subsequent sections unfold as follows: Section “The odd Frechet Half-Logistic (OFHL) model” introduces the Probability Density Function (PDF) and Cumulative Distribution Function (CDF) of the OFHL model, alongside several associated functions. In Section “Basic properties”, the emphasis switches to examining the statistical characteristics such as the Quantile function, moments, moment generating function, conditional and incomplete moments, mean residual time, mean waiting time, as well as minima and maxima associated with the OFHL model. In Section “Parameter estimation of OFHL model”, a comprehensive analysis of six classical point estimation approaches for the OFHL model is thoroughly examined. In Section “Simulation study”, an extensive Monte Carlo simulation analysis is employed to evaluate the performance of these estimators. Finally, Section “Real data applications” showcases two applications using real data, demonstrating the flexibility of the suggested approach. The study concludes in Section “Conclusion”, summarizing the findings and implications of the research.

## The odd Frechet Half-Logistic (OFHL) model

In this part, we investigate the Odd Frechet Half-Logistic model and look at aspects of its statistical properties. By inserting Eqs. (1) and (2) into Eq. (4), we deduce the OFHL PDF having two positive shapes parameters $\alpha \; {\text{and}} \; \theta$, abbreviated as $OFHL\left(\alpha ,\theta \right)$, given by

5 $$f\left(x;\alpha ,\theta \right)=\frac{\alpha \theta {\left(2{e}^{-\theta x}\right)}^{\alpha }}{{\left(1-{e}^{-\theta x}\right)}^{\alpha +1}}exp\left\{-{\left(\frac{2{e}^{-\theta x}}{1-{e}^{-\theta x}}\right)}^{\alpha }\right\} ; x>0,$$

The CDF of the OFHL model corresponding to (5) is computed as

6 $$F\left(x;\alpha ,\theta \right)=exp\left\{-{\left(\frac{2{e}^{-\theta x}}{1-{e}^{-\theta x}}\right)}^{\alpha }\right\} ; x>0$$

### Reliability function

The mathematical expressions for reliability function of $OFHL\left(\alpha ,\theta \right)$ distribution is calculated as

7 $$R\left(x;\alpha ,\theta \right)=1-exp\left\{-{\left(\frac{2{e}^{-\theta x}}{1-{e}^{-\theta x}}\right)}^{\alpha }\right\}$$

### Hazard rate function

The associated hazard rate function of $OFHL\left(\alpha ,\theta \right)$ distribution takes the form

8 $$h\left(x;\alpha ,\theta \right)=\frac{\alpha \theta {\left(2{e}^{-\theta x}\right)}^{\alpha }exp\left\{-{\left(\frac{2{e}^{-\theta x}}{1-{e}^{-\theta x}}\right)}^{\alpha }\right\}}{{\left(1-{e}^{-\theta x}\right)}^{\alpha +1}\left[1-exp\left\{-{\left(\frac{2{e}^{-\theta x}}{1-{e}^{-\theta x}}\right)}^{\alpha }\right\}\right]}.$$

**Figure 1:**
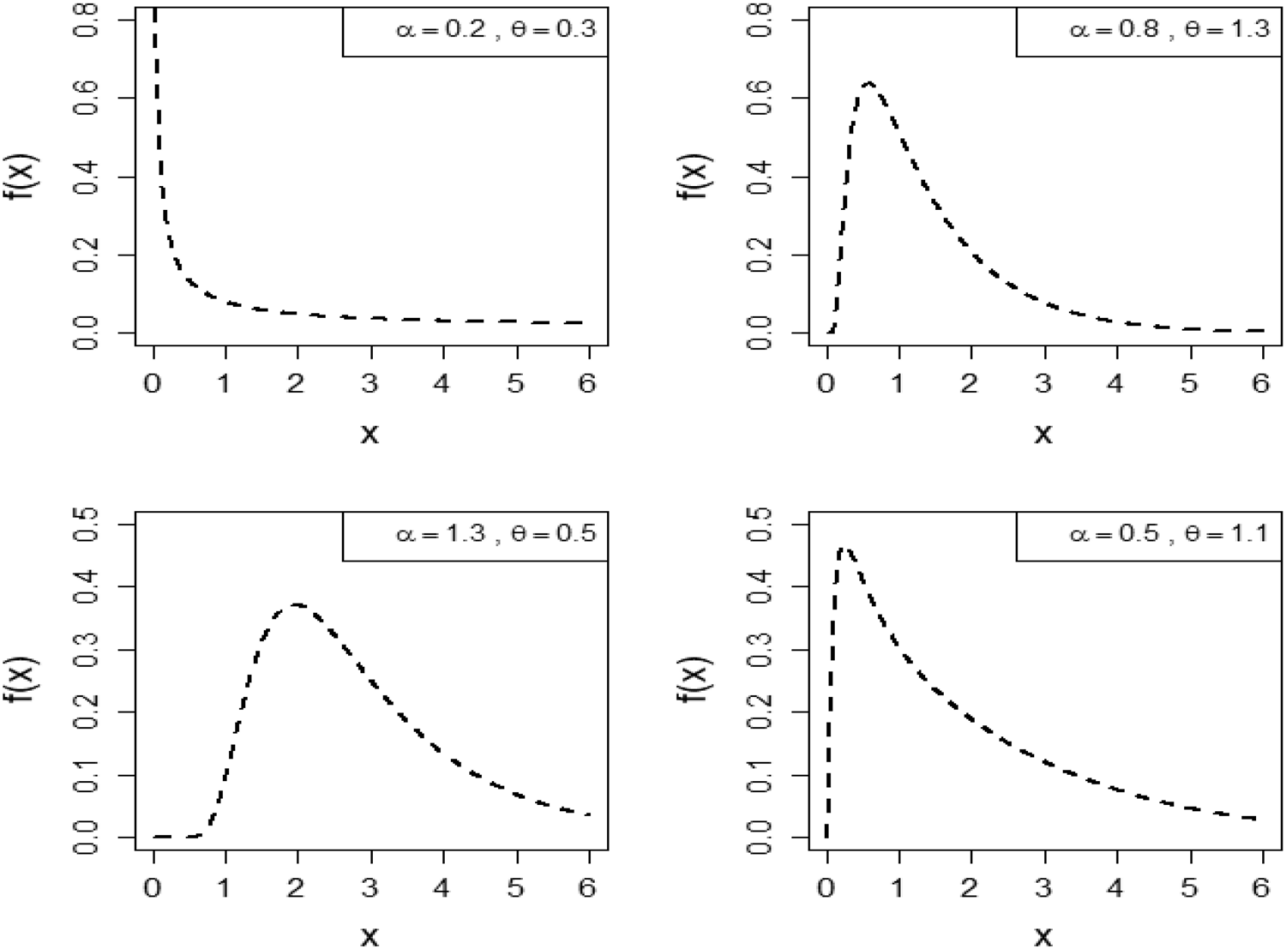
Variations of PDF of $OFHL\left(\alpha ,\theta \right)$ distribution along with parameters $\alpha \; {\text{and}} \; \theta$.

Figure 1 visually illustrates the PDF of OFHL model across various values of parameters $\alpha \; {\text{and}} \; \theta$. This depiction showcases the versatility of the OFHL model, exhibiting PDFs that can be unimodal, right-skewed, symmetric, or even demonstrate an increasing density function. These variations portray the model's adaptability in capturing diverse data patterns related to lifetime distributions.

**Figure 2:**
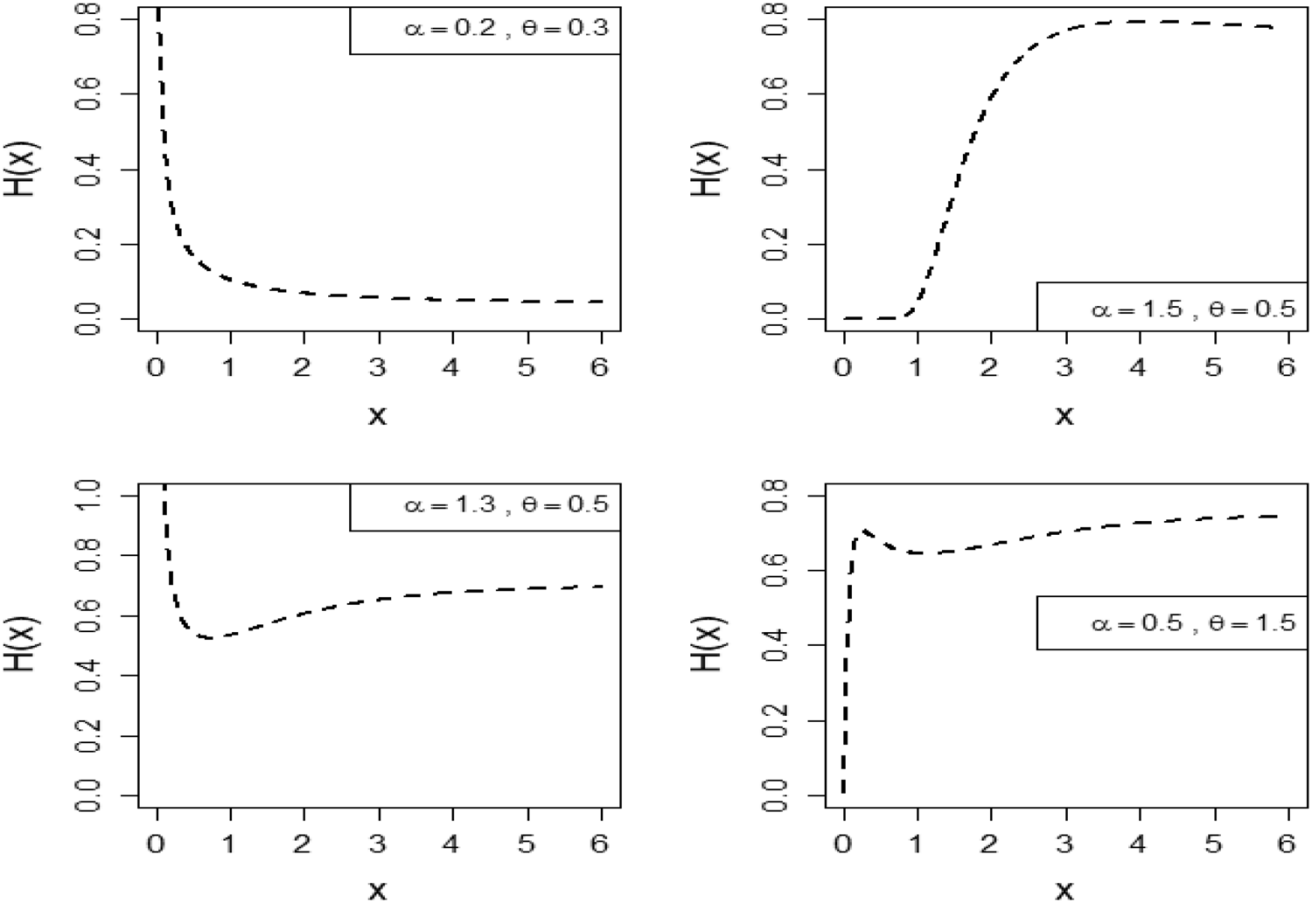
Fluctuations of HRF of $OFHL\left(\alpha ,\theta \right)$ distribution along with parameters $\alpha \; {\text{and}} \; \theta$.

**Figure 3:**
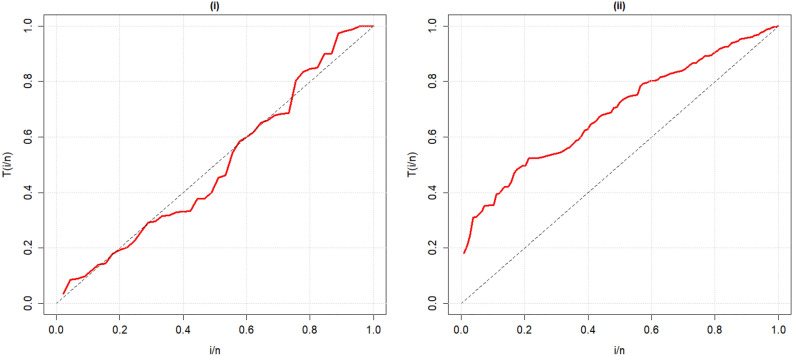
TTT plots of the Covid-19 and Survival time datasets.

Figure 2 depicts the graphical representation HRF for distinct parameter values of the OFHL model. This graphical representation demonstrates the flexibility of the OFHL model in modeling different types of lifetime data by displaying HRFs that can be decreasing, increasing, exhibit a bathtub-shaped pattern, or even an inverted bathtub-shaped pattern. This flexibility highlights the model’s capability to capture a wide range of behaviours observed in lifetime data analysis (Fig. 3).

### Series expansion of the OFHL model

A useful series expansion of the PDF and CDF of the OFHL model is provided in this section.

The series expansion of ${e}^{-y}$ is derived by using Taylor series expansion as given by

9 $${e}^{-y}=\sum_{\xi =0}^{\infty }{\left(-1\right)}^{\xi }\frac{{y}^{\xi }}{\xi !}.$$

The binomial series expansion of ${\left(1-x\right)}^{-n}$ is derived by using the binomial theorem and Taylor series expansion. The expansion is derived as

10 $${\left(1-x\right)}^{-n}=\sum_{k=0}^{\infty }\left(\begin{array}{c}n+k-1\\ k\end{array}\right){x}^{k} , \mathrm{for }\left|{\text{x}}\right|<1,$$

Then, it follows that, using Eq. (9) and Eq. (10), we can expand

11 $$exp\left\{-{\left(\frac{2{e}^{-\theta x}}{1-{e}^{-\theta x}}\right)}^{\alpha }\right\}=\sum_{k=0}^{\infty }\frac{{\left(-1\right)}^{k}}{k!}{\left(\frac{2{e}^{-\theta x}}{1-{e}^{-\theta x}}\right)}^{\alpha k},$$

and

12 $${\left(1-{e}^{-\theta x}\right)}^{-\left(\alpha k+\alpha +1\right)}=\sum_{   \ell    =0}^{\infty }\left(\begin{array}{c}\alpha k+\alpha +   \ell    \\    \ell    \end{array}\right){\left({e}^{-\theta x}\right)}^{   \ell    }$$

Using Eq. (11) and Eq. (12) in Eq. (6), the PDF of the $OFHL\left(\alpha ,\theta \right)$ model can be rewritten as

13 $$ \begin{gathered} f\left( {x;\alpha ,\theta } \right) = \alpha \theta \mathop \sum \limits_{k,\ell = 0}^{\infty } \frac{{\left( { - 1} \right)^{k} }}{k!}\left( {\begin{array}{*{20}c} {\alpha k + \alpha + \ell } \\ \ell \\ \end{array} } \right)2^{{\alpha \left( {k + 1} \right)}} \left( {e^{ - \theta x} } \right)^{{\alpha \left( {k + 1} \right) + \ell }} , \hfill \\ f\left( {x;\alpha ,\theta } \right) = \alpha \theta \mathop \sum \limits_{k,\ell = 0}^{\infty } \eta_{k,\ell } 2^{{\alpha \left( {k + 1} \right)}} \left( {e^{ - \theta x} } \right)^{{\alpha \left( {k + 1} \right) + \ell }} ; x > 0, \alpha ,\theta > 0 \hfill \\ \end{gathered} $$

where

$${\eta }_{k,   \ell    }=\frac{{\left(-1\right)}^{k}}{k!}\left(\begin{array}{c}\alpha k+\alpha +   \ell    \\    \ell    \end{array}\right).$$

## Basic properties

This part is devoted to derive some of the basic properties of the $OFHL\left(\alpha ,\theta \right)$ model such as quantile function, ordinary moments, incomplete and conditional moments and order statistics.

### Quantile function

By inverting the CDF of the $OFHL\left(\alpha ,\theta \right)$ model, the expression for quantile function $Q(u)$ of the random variable $X$ is calculated as

$$Q\left(u\right)=\frac{1}{\theta }log\left[1+\frac{2}{{\left\{-log\left(u\right)\right\}}^{\frac{1}{\alpha }}}\right] .$$

where $u$ is the uniform random variable defined on a unit interval [0,1].

As a result, the median (M) of the $OFHL\left(\alpha ,\theta \right)$ model is computed by setting $u=0.5$, we get

$$M=\frac{1}{\theta }log\left[1+\frac{2}{{\left\{-log\left(0.5\right)\right\}}^{\frac{1}{\alpha }}}\right].$$

### Moments and moment generating function

If $X$ follows $OFHL\left(\alpha ,\theta \right)$ model, then the ${r}{th}$ moment about origin (raw moments) can be evaluated by extending the PDF given by Eq. (13)

$$E\left({X}^{r}\right)=\underset{0}{\overset{\infty }{\int }}{x}^{r}f\left(x;\alpha ,\theta \right)dx ;r=\mathrm{1,2},3,\dots $$

14 $$E\left({X}^{r}\right)=\alpha \theta \sum_{k,   \ell    =0}^{\infty }{\eta }_{k,   \ell    }{2}^{\alpha \left(k+1\right)}\underset{0}{\overset{\infty }{\int }}{x}^{r}{\left({e}^{-\theta x}\right)}^{\alpha \left(k+1\right)+   \ell    }dx$$

Using Integration via substitution method in Eq. (14), we perform the following operations:

Let,

$$\left( {\alpha \left( {k + 1} \right) + \ell } \right)x = z~~~\quad  \Rightarrow x = \frac{z}{{\alpha \left( {k + 1} \right) + \ell }}~~ \Rightarrow ~dx = \frac{{dz}}{{\alpha \left( {k + 1} \right) + \ell }}.$$

Thus,

$$E\left({X}^{r}\right)=\alpha \theta \sum_{k,   \ell    =0}^{\infty }{\eta }_{k,   \ell    }{2}^{\alpha \left(k+1\right)}\underset{0}{\overset{\infty }{\int }}\frac{{z}^{r}{e}^{-\theta z}}{{\left(\alpha \left(k+1\right)+   \ell    \right)}^{r+1}} dz.$$

On simplification, we get

15 $$E\left({X}^{r}\right)=\alpha \sum_{k,   \ell    =0}^{\infty }{\eta }_{k,   \ell    }\frac{{2}^{\alpha \left(k+1\right)}}{{\left(\alpha \left(k+1\right)+   \ell    \right)}^{r+1}}\frac{\Gamma \left(r+1\right)}{{\theta }^{r}}.$$

The moment generating function of $OFHL\left(\alpha ,\theta \right)$ model utilizing the Maclaurin series is mentioned as

$${M}_{X}\left(t\right)=E\left({e}^{tx}\right)=\sum_{r=0}^{\infty }\frac{{t}^{r}}{r!}E\left({X}^{r}\right),$$

Thus, by using Eq. (15), the expression for moment generating function is computed as

$${M}_{X}\left(t\right)=\alpha \sum_{k,   \ell    =0}^{\infty }{\eta }_{k,   \ell    }\frac{{t}^{r}}{r!}\frac{{2}^{\alpha \left(k+1\right)}}{{\left(\alpha \left(k+1\right)+   \ell    \right)}^{r+1}}\frac{\Gamma \left(r+1\right)}{{\theta }^{r}}.$$

### Incomplete and conditional moments

If $X$ belongs to $OFHL\left(\alpha ,\theta \right)$ distribution, then the ${r}{th}$ incomplete moment is given by

$${\varphi }_{r}\left(y\right)=\underset{0}{\overset{y}{\int }}{x}^{r}f\left(x;\alpha ,\theta \right)dx ;r=\mathrm{1,2},3,\dots $$

Using the PDF (13), we can write

$${\varphi }_{r}\left(y\right)==\alpha \theta \sum_{k,   \ell    =0}^{\infty }{\eta }_{k,   \ell    }{2}^{\alpha \left(k+1\right)}\underset{0}{\overset{y}{\int }}{x}^{r}{\left({e}^{-\theta x}\right)}^{\alpha \left(k+1\right)+   \ell    }dx$$

On simplification, we obtain

$${\varphi }_{r}\left(y\right)=\alpha \sum_{k,   \ell    =0}^{\infty }{\eta }_{k,   \ell    }\frac{{2}^{\alpha \left(k+1\right)}}{{\left(\alpha \left(k+1\right)+   \ell    \right)}^{r+1}}\frac{\gamma \left\{r+1,\left(\alpha \left(k+1\right)+   \ell    \right)\theta y\right\}}{{\theta }^{r}}.$$

where, $\gamma \left(a,b\right)=\underset{0}{\overset{a}{\int }}{x}^{b-1}{e}^{-x}dx$ is the lower incomplete gamma function.

Furthermore, the ${r}{th}$ conditional moment, say ${\Delta }_{r}\left(y\right)=E\left({Y}^{r}|Y>y\right)$ is given by

$${\Delta }_{r}\left(y\right)=\frac{1}{\overline{F }(y)}\underset{y}{\overset{\infty }{\int }}{x}^{r}f\left(x;\alpha ,\theta \right)dx ;r=\mathrm{1,2},3,\dots $$

Hence, by using (13), ${\Delta }_{r}\left(y\right)$ can be written as

$${\Delta }_{r}\left(y\right)=\frac{\alpha \theta \sum_{k,   \ell    =0}^{\infty }{\eta }_{k,   \ell    }{2}^{\alpha \left(k+1\right)}\underset{y}{\overset{\infty }{\int }}{x}^{r}{\left({e}^{-\theta x}\right)}^{\alpha \left(k+1\right)+   \ell    }dx}{1-\sum_{k=0}^{\infty }\frac{{\left(-1\right)}^{k}}{k!}{\left(\frac{2{e}^{-\theta x}}{1-{e}^{-\theta x}}\right)}^{\alpha k}}$$

On simplication, the final expression comes out to be

$${\Delta }_{r}\left(y\right)=\frac{\alpha \sum_{k,   \ell    =0}^{\infty }{\eta }_{k,   \ell    }\frac{{2}^{\alpha \left(k+1\right)}}{{\left(\alpha \left(k+1\right)+   \ell    \right)}^{r+1}}\frac{\Gamma \left\{r+1,\left(\alpha \left(k+1\right)+   \ell    \right)\theta y\right\}}{{\theta }^{r}}}{1-\sum_{k=0}^{\infty }\frac{{\left(-1\right)}^{k}}{k!}{\left(\frac{2{e}^{-\theta x}}{1-{e}^{-\theta x}}\right)}^{\alpha k}}.$$

### Mean residual life and mean waiting time

Suppose $X$ is a continuous random variable having survival function $R(x)$, the mean residual life function, say $\pi (t)$ is defined as the expected life of an item after it has reached a certain age $t$, is given

16 $$\pi \left(t\right)=\frac{1}{R(t)}\left(E\left(t\right)-\underset{0}{\overset{t}{\int }}xf\left(x;\alpha ,\theta \right)dx\right)-t$$

where,

17 $$E\left(t\right)=\frac{\alpha }{\theta }\sum_{k,   \ell    =0}^{\infty }{\eta }_{k,   \ell    }\frac{{2}^{\alpha \left(k+1\right)}}{{\left(\alpha \left(k+1\right)+   \ell    \right)}^{2}}$$

and

18 $$\underset{0}{\overset{t}{\int }}xf\left(x;\alpha ,\theta \right)dx=\alpha \sum_{k,   \ell    =0}^{\infty }{\eta }_{k,   \ell    }\frac{{2}^{\alpha \left(k+1\right)}}{{\left(\alpha \left(k+1\right)+   \ell    \right)}^{2}}\gamma \left\{2,\left(\alpha \left(k+1\right)+   \ell    \right)\theta t\right\}$$

By using (7), (17) and (18), $\pi \left(t\right)$ can be written as

$$\pi \left(t\right)=\frac{1}{\left[1-exp\left\{-{\left(\frac{2{e}^{-\theta t}}{1-{e}^{-\theta t}}\right)}^{\alpha }\right\}\right]}\left(\frac{\alpha }{\theta }\sum_{k,   \ell    =0}^{\infty }{\eta }_{k,   \ell    }A-\alpha \sum_{k,   \ell    =0}^{\infty }{\eta }_{k,   \ell    }AB\right)-t$$

where,

$$A=\frac{{2}^{\alpha \left(k+1\right)}}{{\left(\alpha \left(k+1\right)+   \ell    \right)}^{2}},$$

$$B=\gamma \left\{2,\left(\alpha \left(k+1\right)+   \ell    \right)\theta t\right\}.$$

The mean waiting time is very important to analyse the actual time of failure of an already failed item. It represents the amount of time that has passed since an object failed, assuming that the failure occurred within the interval [0, t]. The mean waiting time say $\overline{\pi }(t)$, is defined by

19 $$\overline{\pi }\left(t\right)=t-\frac{1}{F(t)} \underset{0}{\overset{t}{\int }}xf\left(x;\alpha ,\theta \right)dx$$

By using (6) and (18), the final expression of $\overline{\pi }(t)$ comes out to be

$$\overline{\pi }\left(t\right)=t-\frac{\alpha }{exp\left\{-{\left(\frac{2{e}^{-\theta t}}{1-{e}^{-\theta t}}\right)}^{\alpha }\right\}}\sum_{k,   \ell    =0}^{\infty }{\eta }_{k,   \ell    }AB.$$

### Order statistics

In real-world applications incorporating data from life testing studies, order statistics is very important. Assume that ${X}_{1}, {X}_{2}, \dots ,{X}_{n}$ be a random sample with relevant order statistics given by ${X}_{(1)}, {X}_{(2)}, \dots ,{X}_{(n)}$. The CDF of the ${n}{th}$ or maximum order statistics, say ${F}_{n:n}(x)$, is given as

$${F}_{n:n}\left(x\right)={\left[F(x)\right]}^{n}={\left[exp\left\{-{\left(\frac{2{e}^{-\theta x}}{1-{e}^{-\theta x}}\right)}^{\alpha }\right\}\right]}^{n}.$$

Consequently, the PDF of the ${n}{th}$ order statistics, say ${f}_{n:n}(x)$, is computed as

$${f}_{n:n}\left(x\right)=n{\left[F\left(x\right)\right]}^{n-1}f(x)$$

$${f}_{n:n}\left(x\right)=n\alpha \theta {\left[exp\left\{-{\left(\frac{2{e}^{-\theta x}}{1-{e}^{-\theta x}}\right)}^{\alpha }\right\}\right]}^{n-1}\sum_{k,   \ell    =0}^{\infty }{\eta }_{k,   \ell    }{{2}^{\alpha \left(k+1\right)}\left({e}^{-\theta x}\right)}^{\alpha \left(k+1\right)+   \ell    }.$$

The CDF and PDF of minimum order statistics is given by

$${F}_{1:n}\left(x\right)=1-{\left[1-F\left(x\right)\right]}^{n}=1-{\left[1-exp\left\{-{\left(\frac{2{e}^{-\theta x}}{1-{e}^{-\theta x}}\right)}^{\alpha }\right\}\right]}^{n}.$$

and

$${f}_{1:n}\left(x\right)=n\alpha \theta {\left[1-exp\left\{-{\left(\frac{2{e}^{-\theta x}}{1-{e}^{-\theta x}}\right)}^{\alpha }\right\}\right]}^{n-1}\sum_{k,   \ell    =0}^{\infty }{\eta }_{k,   \ell    }{{2}^{\alpha \left(k+1\right)}\left({e}^{-\theta x}\right)}^{\alpha \left(k+1\right)+   \ell    }.$$

## Parameter estimation of OFHL model

This section is devoted to discuss various estimation methodologies for estimating the unknown parameters of the OFHL model, such as the Maximum Likelihood Estimation (MLE), Anderson–Darling Estimation (ADE), Cramer-von Mises Estimation (CVME), Maximum Product of Spacing Estimation (MPSE), Ordinary Least Square Estimation (OLSE) and Weighted Least Square Estimation (WLSE).

### Maximum likelihood estimation

Let ${x}_{1}, {x}_{2}, ,\dots ,{x}_{n}$ be a random sample of size $n$ following OFHL model with parameters $\alpha \; {\text{and}}  \;\theta$, then the logarithmic likelihood function is

20 $$L(\alpha ,\theta )=nlog\left(\alpha \right)+nlog\left(\theta \right)+\alpha \sum_{k=1}^{n}log\left(2{e}^{-\theta {x}_{k}}\right)-\left(\alpha +1\right)\sum_{k=1}^{n}log\left(1-{e}^{-\theta {x}_{k}}\right)-\alpha \sum_{k=1}^{n}\frac{2{e}^{-\theta {x}_{k}}}{\left(1-{e}^{-\theta {x}_{k}}\right)}$$

By differentiating (20) with respect to unknown parameters $\alpha \; {\text{and}} \; \theta$, the resulting partial derivatives are given by

21 $$\frac{\partial L}{\partial \alpha }=\frac{n}{\alpha }+\sum_{k=1}^{n}log\left(2{e}^{-\theta {x}_{k}}\right)-\sum_{k=1}^{n}log\left(1-{e}^{-\theta {x}_{k}}\right)-\sum_{k=1}^{n}\frac{2{e}^{-\theta {x}_{k}}}{\left(1-{e}^{-\theta {x}_{k}}\right)}$$

and

22 $$\frac{\partial L}{\partial \theta }=\frac{n}{\theta }-\alpha \sum_{k=1}^{n}{x}_{k}-\left(\alpha +1\right)\sum_{k=1}^{n}\frac{{x}_{k}{e}^{-\theta {x}_{k}}}{\left(1-{e}^{-\theta {x}_{k}}\right)}+2\alpha \sum_{k=1}^{n}\frac{{x}_{i}{e}^{-\theta {x}_{k}}}{{\left(1-{e}^{-\theta {x}_{k}}\right)}^{2}}$$

By setting the above partial derivatives equal to zero, we could calculate the ML estimators ${\widehat{\alpha }}_{ML} \;{\text{and}} \; {\widehat{\theta }}_{ML}$ of the unknown parameters $\alpha  \, {\text{and}} \; \theta$. Since, the given equations are not in a closed form and cannot be derived analytically. However, R software can be used to get ML estimators of the parameters.

### Anderson darling estimation

The Anderson–Darling test Anderson and Darling^23^ can be used in place of other statistical tests to identify deviations from normality in sample distributions. The AD estimators denoted by ${\widehat{\alpha }}_{AD} \; {\text{and}} \; {\widehat{\theta }}_{AD}$ of the parameters can be evaluated by minimizing the following function with respect to $\alpha \; {\text{and}} \; \theta ,$ respectively

$$A(\alpha ,\theta )=-n-\frac{1}{n}\sum_{k=1}^{n}\left(2k-1\right)\left[{\text{ln}}F\left({x}_{(k)}\right)+{\text{ln}}S\left({x}_{(n+1-k)}\right)\right].$$

$$A(\alpha ,\theta )=-n-\frac{1}{n}\sum_{k=1}^{n}\left(2k-1\right)\left[{\text{ln}}exp\left\{-{\left(\frac{2{e}^{-\theta {x}_{(k)}}}{1-{e}^{-\theta {x}_{(k)}}}\right)}^{\alpha }\right\}+{\text{ln}}\left[1-exp\left\{-{\left(\frac{2{e}^{-\theta {x}_{(k)}}}{1-{e}^{-\theta {x}_{(k)}}}\right)}^{\alpha }\right\}\right]\right].$$

### Cramer-von-Mises estimation

Our choice to utilise minimal distance estimators of the Cramer-von-Mises type was supported by empirical data from Macdonald^24^ proving that the bias of the estimator is smaller than that of the competing minimum distance estimators. The Cramer-von-Mises estimators ${\widehat{\alpha }}_{CVM} \; {\text{and}} \;  {\widehat{\theta }}_{CVM}$ of $\alpha \; {\text{and}} \; \theta$ are derived by minimizing the value of the following function

$$C\left(\alpha ,\theta \right)=\frac{1}{12n}+\sum_{k=1}^{n}{\left\{F\left({x}_{(k)}\right)-\frac{2k-1}{2n}\right\}}^{2}.$$

$$C\left(\alpha ,\theta \right)=\frac{1}{12n}+\sum_{k=1}^{n}{\left[exp\left\{-{\left(\frac{2{e}^{-\theta {x}_{(k)}}}{1-{e}^{-\theta {x}_{(k)}}}\right)}^{\alpha }\right\}-\frac{2k-1}{2n}\right]}^{2}.$$

### Maximum product of spacing estimation

This approach was first developed by Cheng and Amin^25^ as an alternative to ML estimation. The uniform spacing for a random sample of size $n$ taken from the OFHL model can be determined by

$${D}_{k}(\alpha ,\theta )=F\left({x}_{(k)}\right)-F\left({x}_{\left(k-1\right)}\right) ; k=\mathrm{1,2},\dots ,n+1.$$

where ${D}_{k}$ denotes the uniform spacing, $F\left({x}_{(0)}\right)=0 \; {\text{and}} \; F\left({x}_{(n+1)}\right)=1$.

The MPS estimators ${\widehat{\alpha }}_{MPS} \; {\text{and}} \; {\widehat{\theta }}_{MPS}$ of the unknown parameters $\alpha \; {\text{and}}  \; \theta$ can be obtained by maximizing the following function

$$M\left(\alpha ,\theta \right)=\frac{1}{n+1}\sum_{k=1}^{n+1}{\text{log}}{D}_{k}.$$

$$M\left(\alpha ,\theta \right)=\frac{1}{n+1}\sum_{k=1}^{n+1}{\text{log}}\left[exp\left\{-{\left(\frac{2{e}^{-\theta {x}_{(k)}}}{1-{e}^{-\theta {x}_{(k)}}}\right)}^{\alpha }\right\}-exp\left\{-{\left(\frac{2{e}^{-\theta {x}_{(k-1)}}}{1-{e}^{-\theta {x}_{(k-1)}}}\right)}^{\alpha }\right\}\right].$$

### Ordinary and Weighted Least Square Estimation

For estimating the unknown parameters, the least square (LS) and weighted least square (WLS) approaches are widely known Swain et al.^26^. Here, the two approaches for parameter estimation of OFHL model are examined. By minimising the following function with respect to $\alpha\;  {\text{and}}  \;\theta$ respectively, it is possible to obtain the LS and WLS estimators $\widehat{\alpha } \; {\text{and}} \; \widehat{\theta }$ of the OFHL distribution

$$S\left(\alpha ,\theta \right)=\sum_{k=1}^{n}{n}_{k}{\left\{F\left({x}_{(k)}\right)-\frac{i}{n+1}\right\}}^{2}.$$

$$S\left(\alpha ,\theta \right)=\sum_{k=1}^{n}{n}_{k}{\left[exp\left\{-{\left(\frac{2{e}^{-\theta {x}_{(k)}}}{1-{e}^{-\theta {x}_{(k)}}}\right)}^{\alpha }\right\}-\frac{i}{n+1}\right]}^{2}.$$

By setting ${n}_{k}=1$, the LS estimators ${\widehat{\alpha }}_{LS} \; {\text{and}}\;  {\widehat{\theta }}_{LS}$ can be obtained, while as by setting ${n}_{k}=\frac{{(n+1)}^{2}(n+2)}{k(n-k+1)}$ , we can obtain the WLS estimators denoted by ${\widehat{\alpha }}_{WLS} \; {\text{and}} \; {\widehat{\theta }}_{WLS}$.

## Simulation study

It is not theoretically possible to compare the effectiveness of the introduced estimation methods derived in the previous section for estimating the parameters of the OFHL model. Thus, we undertake a Monte Carlo simulation analysis to identify the top estimation method among the six classical estimation methods. In order to do this, we created 1,000 samples at random of sizes 20, 40, 100, 200 and 400 from the OFHL model for three different sets of parameter values, as shown below:

$$Set \, I: \alpha =0.25, \theta =0.75.$$

$$Set \, II: \alpha =1.50, \theta =0.50.$$

$$Set \, III: \alpha =1.25, \theta =1.75.$$

In this simulation study, we evaluate the average values of estimates (AVEs), biases, mean square errors (MSEs) and mean relative errors (MREs). The following mathematical formulas are used to accomplish these objectives:

$$ Bias\left( \vartheta \right) = \frac{1}{N}\mathop \sum \limits_{k = 1}^{N} \left( {\hat{\vartheta } - \vartheta } \right) , MSE\left( \vartheta \right) = \frac{1}{N}\mathop \sum \limits_{k = 1}^{N} \left( {\hat{\vartheta } - \vartheta } \right)^{2} , MSE\left( \vartheta \right) = \frac{1}{N}\mathop \sum \limits_{k = 1}^{N} \left( {\hat{\vartheta } - \vartheta } \right)/\vartheta . $$

where $\vartheta =(\alpha ,\theta )$. All the results related to simulation were obtained by using R-Studio software. The results of the simulation are displayed in Tables 1, 2, 3.

**Table 1:** The Average estimate (AVE), Bias, MSE and MRE for $(\mathrm{\alpha }=0.25,\uptheta =0.75)$.

N	Estimate	Est. Par	MLE	ADE	CVME	MPSE	OLSE	WLSE
20	AVE	$\widehat{\mathrm{\alpha }}$	0.27934	0.26066	0.28885	0.23622	0.26194	0.26514
		$\widehat{\uptheta }$	0.76196	0.80337	0.79912	0.77849	0.83809	0.80793
	Bias	$\widehat{\mathrm{\alpha }}$	0.05421^[3]^	0.05308^[2]^	0.07615^[6]^	0.05062^[1]^	0.07281^[5]^	0.06267^[4]^
		$\widehat{\uptheta }$	0.17949^[1]^	0.22869^[3]^	0.25341^[4]^	0.19821^[2]^	0.30593^[6]^	0.26015^[5]^
	MSE	$\widehat{\mathrm{\alpha }}$	0.00560^[3]^	0.00524^[2]^	0.01320^[6]^	0.00443^[1]^	0.01143^[5]^	0.00857^[4]^
		$\widehat{\uptheta }$	0.05704^[1]^	0.09237^[3]^	0.12542^[4]^	0.06743^[2]^	0.18887^[6]^	0.12258^[5]^
	MRE	$\widehat{\mathrm{\alpha }}$	0.21683^[3]^	0.21234^[2]^	0.30462^[6]^	0.20250^[1]^	0.29122^[5]^	0.25068^[4]^
		$\widehat{\uptheta }$	0.23931^[1]^	0.30491^[3]^	0.33789^[4]^	0.26427^[2]^	0.40791^[6]^	0.34687^[5]^
	$\sum {\text{Ranks}}$		12^[2]^	15^[3]^	30^[5]^	9^[1]^	33^[6]^	27^[4]^
40	AVE	$\widehat{\mathrm{\alpha }}$	0.25642	0.25364	0.26972	0.23952	0.2559	0.26057
		$\widehat{\uptheta }$	0.75775	0.78112	0.7797	0.77144	0.79826	0.77051
	Bias	$\widehat{\mathrm{\alpha }}$	0.03167^[1]^	0.03467^[3]^	0.05169^[6]^	0.03184^[2]^	0.04831^[5]^	0.04282^[4]^
		$\widehat{\uptheta }$	0.12711^[1]^	0.16321^[3]^	0.19627^[6]^	0.12936^[2]^	0.19279^[5]^	0.17052^[4]^
	MSE	$\widehat{\mathrm{\alpha }}$	0.00190^[2]^	0.00202^[3]^	0.00570^[6]^	0.00161^[1]^	0.00437^[5]^	0.00346^[4]^
		$\widehat{\uptheta }$	0.02789^[1]^	0.04677^[3]^	0.07210^[5]^	0.02802^[2]^	0.07444^[6]^	0.04839^[4]^
	MRE	$\widehat{\mathrm{\alpha }}$	0.12667^[1]^	0.13868^[3]^	0.20677^[6]^	0.12736^[2]^	0.19324^[5]^	0.17129^[4]^
		$\widehat{\uptheta }$	0.16948^[1]^	0.21761^[3]^	0.26170^[6]^	0.17248^[2]^	0.25706^[5]^	0.22736^[4]^
	$\sum {\text{Ranks}}$		7^[1]^	18^[3]^	35^[6]^	11^[2]^	31^[5]^	24^[4]^
100	AVE	$\widehat{\mathrm{\alpha }}$	0.25642	0.24924	0.25589	0.24229	0.25073	0.24899
		$\widehat{\uptheta }$	0.74748	0.7725	0.75956	0.765	0.7681	0.77494
	Bias	$\widehat{\mathrm{\alpha }}$	0.01870^[2]^	0.02269^[3]^	0.02687^[5]^	0.01835^[1]^	0.02979^[6]^	0.02325^[4]^
		$\widehat{\uptheta }$	0.07852^[2]^	0.10164^[3]^	0.12311^[6]^	0.07570^[1]^	0.12149^[5]^	0.10625^[4]^
	MSE	$\widehat{\mathrm{\alpha }}$	0.00062^[2]^	0.00079^[3]^	0.00127^[5]^	0.00051^[1]^	0.00145^[6]^	0.00088^[4]^
		$\widehat{\uptheta }$	0.01020^[2]^	0.01753^[3]^	0.02407^[5]^	0.00960^[1]^	0.02413^[6]^	0.01756^[4]^
	MRE	$\widehat{\mathrm{\alpha }}$	0.07481^[2]^	0.09074^[3]^	0.10748^[5]^	0.07338^[1]^	0.11916^[6]^	0.09298^[4]^
		$\widehat{\uptheta }$	0.10469^[2]^	0.13552^[3]^	0.16414^[6]^	0.10094^[1]^	0.16199^[5]^	0.14167^[4]^
	$\sum {\text{Ranks}}$		12^[2]^	18^[3]^	32^[5]^	6^[1]^	34^[6]^	24^[4]^
200	AVE	$\widehat{\mathrm{\alpha }}$	0.25371	0.25188	0.25428	0.24696	0.25114	0.2512
		$\widehat{\uptheta }$	0.74875	0.75315	0.74815	0.7508	0.75334	0.75746
	Bias	$\widehat{\mathrm{\alpha }}$	0.01333^[2]^	0.01552^[3]^	0.02143^[6]^	0.01298^[1]^	0.02008^[5]^	0.01619^[4]^
		$\widehat{\uptheta }$	0.05580^[1]^	0.07406^[4]^	0.08269^[6]^	0.05696^[2]^	0.08221^[5]^	0.07035^[3]^
	MSE	$\widehat{\mathrm{\alpha }}$	0.00029^[1]^	0.00039^[3]^	0.00076^[6]^	0.00028^[2]^	0.00067^[5]^	0.00042^[4]^
		$\widehat{\uptheta }$	0.00513^[2]^	0.00848^[4]^	0.01058^[6]^	0.00506^[1]^	0.01056^[5]^	0.00795^[3]^
	MRE	$\widehat{\mathrm{\alpha }}$	0.05333^[2]^	0.06209^[3]^	0.08570^[6]^	0.05191^[1]^	0.08030^[5]^	0.06478^[4]^
		$\widehat{\uptheta }$	0.07440^[1]^	0.09874^[4]^	0.11025^[6]^	0.07595^[2]^	0.10961^[5]^	0.09381^[3]^
	$\sum {\text{Ranks}}$		9^[1.5]^	21^[3.5]^	36^[6]^	9^[1.5]^	30^[5]^	21^[3.5]^
400	AVE	$\widehat{\mathrm{\alpha }}$	0.25101	0.25024	0.25076	0.2469	0.25057	0.24933
		$\widehat{\uptheta }$	0.75125	0.75255	0.75762	0.75292	0.75183	0.75655
	Bias	$\widehat{\mathrm{\alpha }}$	0.00874^[1]^	0.01088^[3]^	0.01337^[6]^	0.00964^[2]^	0.01250^[5]^	0.01102^[4]^
		$\widehat{\uptheta }$	0.03822^[2]^	0.05070^[3]^	0.05889^[6]^	0.03751^[1]^	0.05582^[5]^	0.05089^[4]^
	MSE	$\widehat{\mathrm{\alpha }}$	0.00012^[1]^	0.00019^[3.5]^	0.00029^[6]^	0.00014^[2]^	0.00026^[5]^	0.00019^[3.5]^
		$\widehat{\uptheta }$	0.00224^[1.5]^	0.00396^[3]^	0.00562^[6]^	0.00224^[1.5]^	0.00490^[5]^	0.00417^[4]^
	MRE	$\widehat{\mathrm{\alpha }}$	0.03495^[1]^	0.04353^[3]^	0.05347^[6]^	0.03855^[2]^	0.05000^[5]^	0.04407^[4]^
		$\widehat{\uptheta }$	0.05096^[2]^	0.06760^[3]^	0.07851^[6]^	0.05001^[1]^	0.07443^[5]^	0.06786^[4]^
	$\sum {\text{Ranks}}$		8.5^[1]^	18.5^[3]^	36^[6]^	9.5^[2]^	30^[5]^	23.5^[4]^

**Table 2:** The Average estimate (AVE), Bias, MSE and MRE for $(\mathrm{\alpha }=1.50,\uptheta =0.50)$.

N	Estimate	Est. Par	MLE	ADE	CVME	MPSE	OLSE	WLSE
20	AVE	$\widehat{\mathrm{\alpha }}$	1.62349	1.53249	1.64023	1.40593	1.50013	1.5177
		$\widehat{\uptheta }$	0.49953	0.50111	0.4999	0.50296	0.50502	0.50123
	Bias	$\widehat{\mathrm{\alpha }}$	0.25228^[3]^	0.23576^[2]^	0.30452^[6]^	0.22689^[1]^	0.27849^[5]^	0.25393^[4]^
		$\widehat{\uptheta }$	0.03807^[4]^	0.03798^[3]^	0.03919^[6]^	0.03685^[1]^	0.03772^[3]^	0.03808^[5]^
	MSE	$\widehat{\mathrm{\alpha }}$	0.11973^[4]^	0.09503^[2]^	0.19730^[6]^	0.07625^[1]^	0.13046^[5]^	0.11330^[3]^
		$\widehat{\uptheta }$	0.00232^[5]^	0.00225^[4]^	0.00240^[6]^	0.00217^[1]^	0.00224^[2.5]^	0.00224^[2.5]^
	MRE	$\widehat{\mathrm{\alpha }}$	0.16819^[3]^	0.15718^[2]^	0.20301^[6]^	0.15126^[1]^	0.18566^[5]^	0.16929^[4]^
		$\widehat{\uptheta }$	0.07615^[4.5]^	0.07595^[3]^	0.07838^[6]^	0.07369^[1]^	0.07543^[2]^	0.07615^[4.5]^
	$\sum {\text{Ranks}}$		23.5^[5]^	16^[2]^	36^[6]^	6^[1]^	22.5^[3]^	23^[4]^
40	AVE	$\widehat{\mathrm{\alpha }}$	1.55929	1.5144	1.56286	1.42963	1.49823	1.51317
		$\widehat{\uptheta }$	0.50039	0.50083	0.49988	0.50269	0.50208	0.50196
	Bias	$\widehat{\mathrm{\alpha }}$	0.14944^[1]^	0.16213^[3]^	0.19976^[6]^	0.15444^[2]^	0.19401^[5]^	0.16325^[4]^
		$\widehat{\uptheta }$	0.02664^[2]^	0.02711^[3]^	0.02747^[4]^	0.02626^[1]^	0.02799^[6]^	0.02748^[5]^
	MSE	$\widehat{\mathrm{\alpha }}$	0.03618^[2]^	0.04276^[3]^	0.06258^[6]^	0.03613^[1]^	0.06100^[5]^	0.04591^[4]^
		$\widehat{\uptheta }$	0.00113^[2]^	0.00119^[4.5]^	0.00119^[4.5]^	0.00110^[1]^	0.00127^[6]^	0.00118^[3]^
	MRE	$\widehat{\mathrm{\alpha }}$	0.09963^[1]^	0.10809^[3]^	0.13317^[6]^	0.10296^[2]^	0.12934^[5]^	0.10884^[4]^
		$\widehat{\uptheta }$	0.05328^[2]^	0.05422^[3]^	0.05494^[4]^	0.05251^[1]^	0.05597^[6]^	0.05495^[5]^
	$\sum {\text{Ranks}}$		10^[2]^	19.5^[3]^	30.5^[5]^	8^[1]^	33^[6]^	25^[4]^
100	AVE	$\widehat{\mathrm{\alpha }}$	1.52698	1.51098	1.52192	1.45496	1.49071	1.50306
		$\widehat{\uptheta }$	0.49885	0.50164	0.49972	0.50106	0.49972	0.50042
	Bias	$\widehat{\mathrm{\alpha }}$	0.09570^[1]^	0.10035^[4]^	0.12095^[6]^	0.09584^[2]^	0.11285^[5]^	0.09964^[3]^
		$\widehat{\uptheta }$	0.01690^[3]^	0.01739^[4]^	0.01751^[5]^	0.01659^[1]^	0.01802^[6]^	0.01683^[2]^
	MSE	$\widehat{\mathrm{\alpha }}$	0.01538^[2]^	0.01633^[4]^	0.02359^[6]^	0.01409^[1]^	0.02063^[5]^	0.01594^[3]^
		$\widehat{\uptheta }$	0.00046^[3]^	0.00048^[4.5]^	0.00048^[4.5]^	0.00043^[1.5]^	0.00052^[6]^	0.00043^[1.5]^
	MRE	$\widehat{\mathrm{\alpha }}$	0.06380^[1]^	0.06690^[4]^	0.08063^[6]^	0.06389^[2]^	0.07523^[5]^	0.06643^[3]^
		$\widehat{\uptheta }$	0.03380^[3]^	0.03479^[4]^	0.03503^[5]^	0.03318^[1]^	0.03604^[6]^	0.03366^[2]^
	$\sum {\text{Ranks}}$		13^[2]^	24.5^[4]^	32.5^[5]^	8.5^[1]^	33^[6]^	14.5^[3]^
200	AVE	$\widehat{\mathrm{\alpha }}$	1.51148	1.50583	1.50771	1.46873	1.50161	1.50229
		$\widehat{\uptheta }$	0.50033	0.50025	0.50009	0.50042	0.50054	0.50021
	Bias	$\widehat{\mathrm{\alpha }}$	0.06230^[1]^	0.06676^[2]^	0.07949^[5]^	0.06813^[3]^	0.08134^[6]^	0.07380^[4]^
		$\widehat{\uptheta }$	0.01150^[1]^	0.01240^[4.5]^	0.01240^[4.5]^	0.01214^[3]^	0.01271^[6]^	0.01196^[2]^
	MSE	$\widehat{\mathrm{\alpha }}$	0.00620^[1]^	0.00730^[3]^	0.00978^[5]^	0.00719^[2]^	0.01047^[6]^	0.00868^[4]^
		$\widehat{\uptheta }$	0.00021^[1]^	0.00024^[4.5]^	0.00024^[4.5]^	0.00022^[2]^	0.00025^[6]^	0.00023^[3]^
	MRE	$\widehat{\mathrm{\alpha }}$	0.04153^[1]^	0.04451^[2]^	0.05299^[5]^	0.04542^[3]^	0.05423^[6]^	0.04920^[4]^
		$\widehat{\uptheta }$	0.02300^[1]^	0.02480^[5]^	0.02479^[4]^	0.02427^[3]^	0.02542^[6]^	0.02392^[2]^
	$\sum {\text{Ranks}}$		6^[1]^	21^[4]^	28^[5]^	16^[2]^	36^[6]^	19^[3]^
400	AVE	$\widehat{\mathrm{\alpha }}$	1.50391	1.5018	1.51058	1.4853	1.49614	1.50174
		$\widehat{\uptheta }$	0.49991	0.50015	0.50002	0.50034	0.49969	0.50029
	Bias	$\widehat{\mathrm{\alpha }}$	0.04345^[1]^	0.04906^[4]^	0.05857^[6]^	0.04716^[2]^	0.05510^[5]^	0.04813^[3]^
		$\widehat{\uptheta }$	0.00844^[2.5]^	0.00848^[4]^	0.00909^[6]^	0.00838^[1]^	0.00889^[5]^	0.00844^[2.5]^
	MSE	$\widehat{\mathrm{\alpha }}$	0.00293^[1]^	0.00376^[4]^	0.00555^[6]^	0.00343^[2]^	0.00486^[5]^	0.00362^[3]^
		$\widehat{\uptheta }$	0.00011^[2.5]^	0.00011^[2.5]^	0.00013^[6]^	0.00011^[2.5]^	0.00012^[5]^	0.00011^[2.5]^
	MRE	$\widehat{\mathrm{\alpha }}$	0.02897^[1]^	0.03271^[4]^	0.03905^[6]^	0.03144^[2]^	0.03673^[5]^	0.03209^[3]^
		$\widehat{\uptheta }$	0.01688^[2.5]^	0.01697^[4]^	0.01819^[6]^	0.01676^[1]^	0.01777^[5]^	0.01688^[2.5]^
	$\sum {\text{Ranks}}$		10.5^[1.5]^	22.5^[4]^	36^[6]^	10.5^[1.5]^	30^[5]^	16.5^[3]^

**Table 3:** The Average estimate (AVE), Bias, MSE and MRE for $(\mathrm{\alpha }=1.25,\uptheta =1.25)$.

N	Estimate	Est. Par	MLE	ADE	CVME	MPSE	OLSE	WLSE
20	AVE	$\widehat{\mathrm{\alpha }}$	1.37061	1.28851	1.36593	1.15531	1.24319	1.24854
		$\widehat{\uptheta }$	1.24745	1.25617	1.24406	1.27439	1.26248	1.27121
	Bias	$\widehat{\mathrm{\alpha }}$	0.21585^[3]^	0.19458^[1]^	0.25103^[6]^	0.19763^[2]^	0.23899^[5]^	0.21820^[4]^
		$\widehat{\uptheta }$	0.10813^[2]^	0.11276^[1]^	0.11641^[6]^	0.10470^[1]^	0.11486^[5]^	0.10897^[3]^
	MSE	$\widehat{\mathrm{\alpha }}$	0.08238^[4]^	0.06516^[2]^	0.12191^[6]^	0.05684^[1]^	0.09416^[5]^	0.07952^[3]^
		$\widehat{\uptheta }$	0.01820^[2]^	0.02000^[4]^	0.02096^[5]^	0.01678^[1]^	0.02149^[6]^	0.01899^[3]^
	MRE	$\widehat{\mathrm{\alpha }}$	0.17268^[4]^	0.15566^[1]^	0.20083^[6]^	0.15811^[2]^	0.19119^[5]^	0.17456^[3]^
		$\widehat{\uptheta }$	0.08650^[2]^	0.09021^[4]^	0.09312^[6]^	0.08376^[1]^	0.09189^[5]^	0.08718^[3]^
	$\sum {\text{Ranks}}$		17^[3]^	13^[2]^	35^[6]^	8^[1]^	31^[5]^	19^[4]^
40	AVE	$\widehat{\mathrm{\alpha }}$	1.28907	1.27342	1.2989	1.19092	1.23236	1.26674
		$\widehat{\uptheta }$	1.25632	1.25108	1.24429	1.25764	1.26492	1.24992
	Bias	$\widehat{\mathrm{\alpha }}$	0.11824^[1]^	0.13451^[2]^	0.16051^[6]^	0.13735^[3]^	0.15870^[5]^	0.13838^[4]^
		$\widehat{\uptheta }$	0.07812^[2]^	0.08437^[6]^	0.07991^[4]^	0.07543^[1]^	0.08425^[5]^	0.07906^[3]^
	MSE	$\widehat{\mathrm{\alpha }}$	0.02309^[1]^	0.03126^[3]^	0.04649^[6]^	0.02836^[2]^	0.04595^[5]^	0.03183^[4]^
		$\widehat{\uptheta }$	0.00970^[3]^	0.01112^[5]^	0.00993^[4]^	0.00915^[1]^	0.01128^[6]^	0.00941^[2]^
	MRE	$\widehat{\mathrm{\alpha }}$	0.09459^[1]^	0.10761^[3]^	0.12841^[6]^	0.10988^[4]^	0.12696^[1]^	0.11071^[5]^
		$\widehat{\uptheta }$	0.06250^[2]^	0.06749^[6]^	0.06393^[4]^	0.06034^[1]^	0.06740^[5]^	0.06325^[3]^
	$\sum {\text{Ranks}}$		10^[1]^	25^[4]^	30^[6]^	12^[2]^	27^[5]^	21^[3]^
100	AVE	$\widehat{\mathrm{\alpha }}$	1.26636	1.25698	1.27696	1.21888	1.25057	1.25582
		$\widehat{\uptheta }$	1.2525	1.24805	1.24939	1.25629	1.25499	1.25306
	Bias	$\widehat{\mathrm{\alpha }}$	0.07358^[1]^	0.07645^[2]^	0.09298^[5]^	0.07789^[3]^	0.09482^[6]^	0.08729^[4]^
		$\widehat{\uptheta }$	0.05055^[3]^	0.04709^[1]^	0.05459^[6]^	0.04713^[2]^	0.05310^[5]^	0.05112^[4]^
	MSE	$\widehat{\mathrm{\alpha }}$	0.00853^[1]^	0.00912^[2]^	0.01405^[5]^	0.00932^[3]^	0.01467^[6]^	0.01212^[4]^
		$\widehat{\uptheta }$	0.00385^[3]^	0.00345^[1]^	0.00455^[6]^	0.00365^[2]^	0.00453^[5]^	0.00394^[4]^
	MRE	$\widehat{\mathrm{\alpha }}$	0.05886^[1]^	0.06116^[2]^	0.07438^[5]^	0.06231^[3]^	0.07585^[6]^	0.06983^[4]^
		$\widehat{\uptheta }$	0.04044^[3]^	0.03767^[1]^	0.04367^[6]^	0.03770^[2]^	0.04248^[5]^	0.04090^[4]^
	$\sum {\text{Ranks}}$		12^[2]^	9^[1]^	33^[5.5]^	15^[3]^	33^[5.5]^	24^[4]^
200	AVE	$\widehat{\mathrm{\alpha }}$	1.25682	1.25811	1.26529	1.22585	1.24223	1.25166
		$\widehat{\uptheta }$	1.24846	1.25095	1.25405	1.25419	1.25016	1.25095
	Bias	$\widehat{\mathrm{\alpha }}$	0.05402 ^[2]^	0.05631^[4]^	0.06925^[6]^	0.05374^[1]^	0.06119^[5]^	0.05565^[3]^
		$\widehat{\uptheta }$	0.03368^[1]^	0.03789^[6]^	0.03781^[5]^	0.03449^[2]^	0.03689^[4]^	0.03538^[3]^
	MSE	$\widehat{\mathrm{\alpha }}$	0.00467^[2]^	0.00504^[4]^	0.00778^[6]^	0.00446^[1]^	0.00588^[5]^	0.00501^[3]^
		$\widehat{\uptheta }$	0.00175^[1]^	0.00222^[6]^	0.00220^[5]^	0.00186^[2]^	0.00209^[4]^	0.00193^[3]^
	MRE	$\widehat{\mathrm{\alpha }}$	0.04321^[2]^	0.04505^[4]^	0.05540^[6]^	0.04299^[1]^	0.04895^[5]^	0.04452^[3]^
		$\widehat{\uptheta }$	0.02694^[1]^	0.03031^[6]^	0.03025^[5]^	0.02759^[2]^	0.02951^[4]^	0.02831^[3]^
	$\sum {\text{Ranks}}$		9^[1.5]^	30^[5]^	33^[6]^	9^[1.5]^	27^[4]^	18^[3]^
400	AVE	$\widehat{\mathrm{\alpha }}$	1.25809	1.25388	1.25603	1.23663	1.25625	1.254
		$\widehat{\uptheta }$	1.25261	1.25165	1.24691	1.25091	1.24915	1.2503
	Bias	$\widehat{\mathrm{\alpha }}$	0.03746^[1]^	0.03969^[3]^	0.04717^[5]^	0.03891^[2]^	0.04930^[6]^	0.04197^[4]^
		$\widehat{\uptheta }$	0.02442^[1]^	0.02517^[3]^	0.02624^[6]^	0.02462^[2]^	0.02590^[5]^	0.02581^[4]^
	MSE	$\widehat{\mathrm{\alpha }}$	0.00223^[1]^	0.00250^[3]^	0.00358^[5]^	0.00225^[2]^	0.00387^[6]^	0.00283^[4]^
		$\widehat{\uptheta }$	0.00096^[2]^	0.00100^[3]^	0.00107^[6]^	0.00095^[1]^	0.00106^[4.5]^	0.00106^[4.5]^
	MRE	$\widehat{\mathrm{\alpha }}$	0.02997^[1]^	0.03175^[3]^	0.03774^[6]^	0.03113^[2]^	0.03944^[4]^	0.03358^[5]^
		$\widehat{\uptheta }$	0.01954^[1]^	0.02014^[3]^	0.02100^[6]^	0.01970^[2]^	0.02072^[5]^	0.02065^[4]^
	$\sum {\text{Ranks}}$		7^[1]^	18^[3]^	34^[6]^	11^[2]^	30.5^[5]^	25.5^[4]^

## Interpretations at the end of the simulation Results

- The absolute biases of $\widehat{\alpha }  \; {\text{and}} \; \widehat{\theta }$ decreases as $n$ rises under all estimation techniques.
- As $n$ increases, the MSE reduces for all the methods of estimation, satisfying the consistency criteria.
- In all estimation procedures, as $n$ increases, the discrepancy between estimates and specified parameters decreases.
- In terms of MSE, the method of maximum product of spacing estimation outperforms the other methods in the majority of situations.
- In light of our analysis and from Table 4, we determine that MPSE performs best (overall score of 23.5) as n approaches infinity in terms of bias, MSE and MRE for the parameter combinations taken into account in our study.
- In most instances, the second best performing estimator is MLE (overall score of 27.5) followed by ADE (overall score of 47.5).
- The overall position of the remaining estimators is displayed in Table 4.

**Table 4:** Partial and overall rankings of all estimation methodologies.

Parameter	n	MLE	ADE	CVME	MPSE	OLSE	WLSE
$\mathrm{\alpha }=0.25,\uptheta =0.75$	20	2	3	6	1	6	4
	40	1	3	6	2	5	4
	100	2	3	5	1	6	4
	200	1.5	3.5	6	1.5	5	3.5
	400	1	3	6	2	5	4
$\mathrm{\alpha }=1.50,\uptheta =0.50$	20	5	2	6	1	3	4
	40	2	3	5	1	6	4
	100	2	4	5	1	6	3
	200	1	4	5	2	6	3
	400	1.5	4	6	1.5	5	3
$\mathrm{\alpha }=1.25,\uptheta =1.25$	20	3	2	6	1	5	4
	40	1	4	6	2	5	3
	100	2	1	5.5	3	5.5	4
	200	1.5	5	6	1.5	4	3
	400	1	3	6	2	5	4
$\sum {\text{Ranks}}$		27.5	47.5	85.5	23.5	77.5	54.5
Overall Rank		2	3	6	1	5	4

The overall inference drawn from the simulation results is that as sample size increases, bias, MSE and MRE for all parameters goes on decreasing and eventually will reach to zero. This shows the precision of both the numerical computations for the OFHL parameters and the estimation techniques.

## Real data applications

To highlight the significance of the OFHL distribution discussed in Section “The odd Frechet Half-Logistic (OFHL) model”, we demonstrate two real applications to assess the adaptability of the subjected model.

**Dataset I:** The first dataset covers 108 days from 4 March to 20 July 2020 and corresponds to the COVID-19 mortality rate for Mexico. It was previously examined by Almongy et al.^27^. The values of first data: 8.826, 6.105, 10.383, 7.267, 13.220, 6.015, 10.855, 6.122, 10.685, 10.035, 5.242, 7.630, 14.604, 7.903, 6.327, 9.391, 14.962, 4.730, 3.215, 16.498, 11.665, 9.284, 12.878, 6.656, 3.440, 5.854, 8.813, 10.043, 7.260, 5.985, 4.424, 4.344, 5.143, 9.935, 7.840, 9.550, 6.968, 6.370, 3.537, 3.286, 10.158, 8.108, 6.697, 7.151, 6.560, 2.988, 3.336, 6.814, 8.325, 7.854, 8.551, 3.228, 3.499, 3.751, 7.486, 6.625, 6.140, 4.909, 4.661, 1.867, 2.838, 5.392, 12.042, 8.696, 6.412, 3.395, 1.815, 3.327, 5.406, 6.182, 4.949, 4.089, 3.359, 2.070, 3.298, 5.317, 5.442, 4.557, 4.292, 2.500, 6.535, 4.648, 4.697, 5.459, 4.120, 3.922, 3.219, 1.402, 2.438, 3.257, 3.632, 3.233, 3.027, 2.352, 1.205, 2.077, 3.778, 3.218, 2.926, 2.601, 2.065, 1.041, 1.800, 3.029, 2.058, 2.326, 2.506, 1.923.

**Dataset II:** The second dataset used in this study corresponds to the survival periods (measured in years) of 46 patients treated with chemotherapy only. The earlier reports of this dataset were made by Bekker et al.^28^ and Fulment et al.^29^.The values of second are**:** 0.047, 0.115, 0.121, 0.132, 0.164, 0.197, 0.203, 0.260, 0.282, 0.296, 0.334, 0.395, 0.458, 0.466, 0.501, 0.507, 0.529, 0.534, 0.540, 0.641, 0.644, 0.696, 0.841, 0.863, 1.099, 1.219, 1.271, 1.326, 1.447, 1.485, 1.553, 1.581, 1.589, 2.178, 2.343, 2.416, 2.444, 2.825, 2.830, 3.578, 3.658, 3.743, 3.978, 4.003, 4.033.

For the purposes of comparison, the OFHL model is contrasted with popular half-logistic extensions, such as Kumaraswamy half-logistic (KHL), exponentiated half-logistic (EHL), Marshal-Olkin half-logistic (MOHL), power half-logistic (PHL), and half-logistic (HL) distribution. The MLEs of the model parameters and the goodness-of-fit (GoF) metrics, including Akaike Information (AIC), Schwarz Information (SIC), Consistent Akaike Information (CAIC), and Hannan-Quinn Information (HQIC) criteria, are shown in Tables 5 and 6. The best match for the actual dataset may be the model having the least quantities of the aforementioned GoF metrics. For the investigated distributions, we also assess the Anderson–Darling (A^*^), Cramer-Von Mises (W^*^), Kolmogorov–Smirnov (K–S) statistic and associated *P*-value (PV).

**Table 5:** The MLEs and GoF metrics for Covid-19 dataset.

Model	MLEs	AIC	SIC	CAIC	HQIC	A*	W*	KS (*p*-value)
OFHL	$\widehat{\mathrm{\alpha }}=1.0083$ $\widehat{\uptheta }=0.2807$	536.9064	542.2706	537.0206	539.0814	0.3623	0.0622	0.0659 (0.7316)
KHL	$\widehat{\mathrm{\alpha }}=4.1539$ $\widehat{\upbeta }=0.4101$ $\widehat{\uptheta }=0.7520$	538.6304	546.6768	538.8611	541.8929	0.3696	0.0652	0.0696 (0.6758)
EHL	$\widehat{\mathrm{\alpha }}=2.6444$ $\widehat{\uptheta }=0.3941$	537.9374	543.3016	538.0517	540.1124	0.4242	0.0733	0.0807 (0.4824)
MOHL	$\widehat{\mathrm{\alpha }}=5.6637$ $\widehat{\uptheta }=0.4716$	548.9523	554.3166	549.0666	551.1274	1.0985	0.1729	0.0778 (0.5307)
PHL	$\widehat{\mathrm{\alpha }}=3.7335$ $\widehat{\uptheta }=0.4397$	544.1177	549.482	544.232	546.2927	0.7854	0.1256	0.0883 (0.3679)
HL	$\widehat{\uptheta }=0.2539$	573.5781	576.2602	573.6158	574.6656	0.5124	0.0839	0.1969 (0.0014)

**Table 6:** The MLEs and GoF metrics statistics for Survival time dataset.

Model	MLEs	AIC	SIC	CAIC	HQIC	A*	W*	KS (*p*-value)
OFHL	$\widehat{\mathrm{\alpha }}=0.4835$ $\widehat{\uptheta }=1.7273$	117.6707	121.2840	117.9564	119.0177	0.3646	0.0515	0.1005 (0.7155)
KHL	$\widehat{\mathrm{\alpha }}=0.9908$ $\widehat{\upbeta }=6.4098$ $\widehat{\uptheta }=0.2035$	122.1589	127.5789	122.7442	124.1794	0.5803	0.0872	0.1116 (0.5905)
EHL	$\widehat{\mathrm{\alpha }}=0.8617$ $\widehat{\uptheta }=0.9243$	121.7464	125.3597	122.0321	123.0934	0.6964	0.1061	0.1252 (0.4453)
MOHL	$\widehat{\mathrm{\alpha }}=0.5095$ $\widehat{\uptheta }=0.7523$	120.4358	124.0491	120.7215	121.7828	0.5319	0.0794	0.0926 (0.8018)
PHL	$\widehat{\mathrm{\alpha }}=-1.268$ $\widehat{\uptheta }=0.7631$	120.7452	124.3585	121.0309	122.0922	0.5637	0.0846	0.1061 (0.6524)
HL	$\widehat{\uptheta }=1.0012$	120.4103	122.2170	120.5033	121.0838	0.6958	0.1061	0.1583 (0.1882)

**Figure 4:**
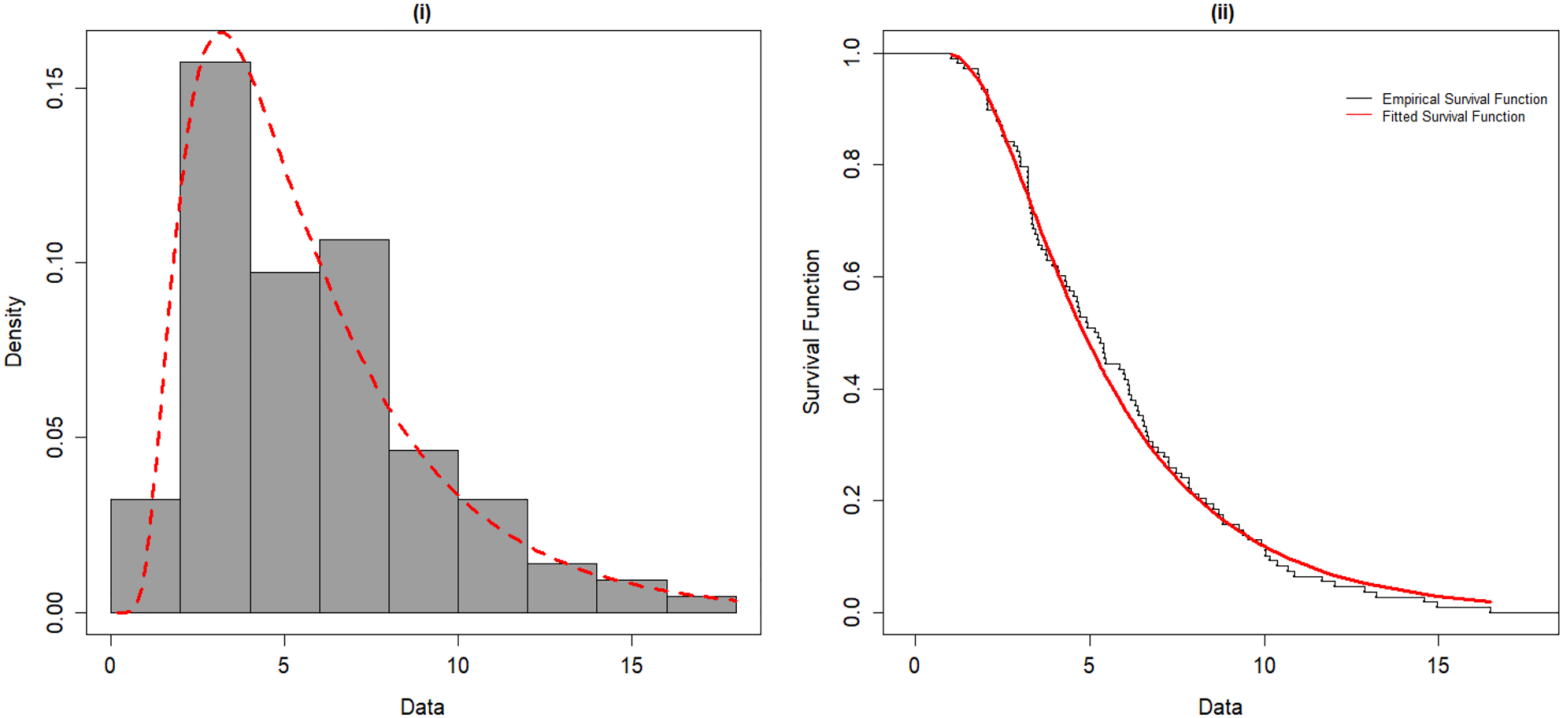
(**i**) Fitted density plot of OFHL distribution. (**ii**) The fitted survival plot and empirical survival plot of OFHL for Covid-19 dataset.

**Figure 5:**
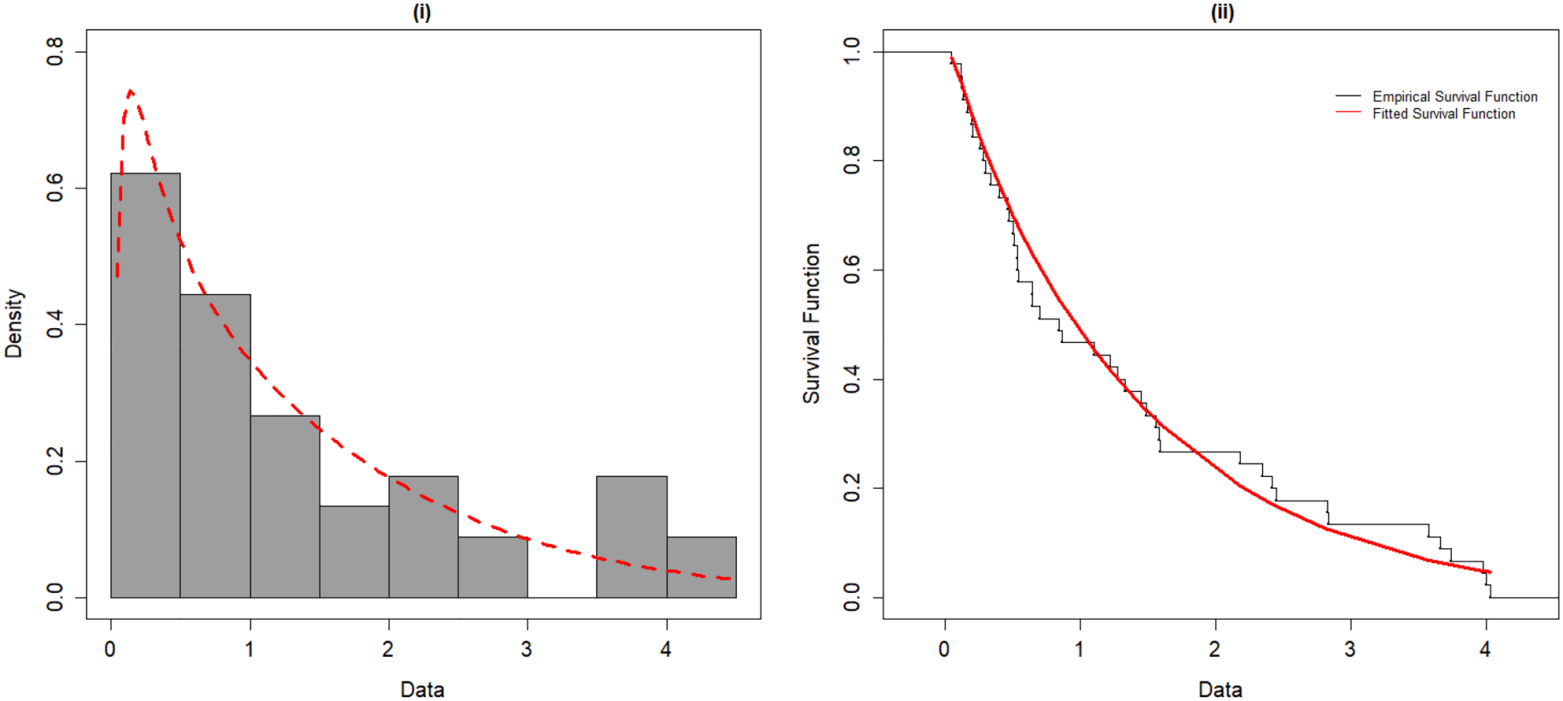
(i) Fitted density plot of OFHL distribution. (ii) The fitted survival plot and empirical survival plot of OFHL for Survival time dataset.

**Figure 6:**
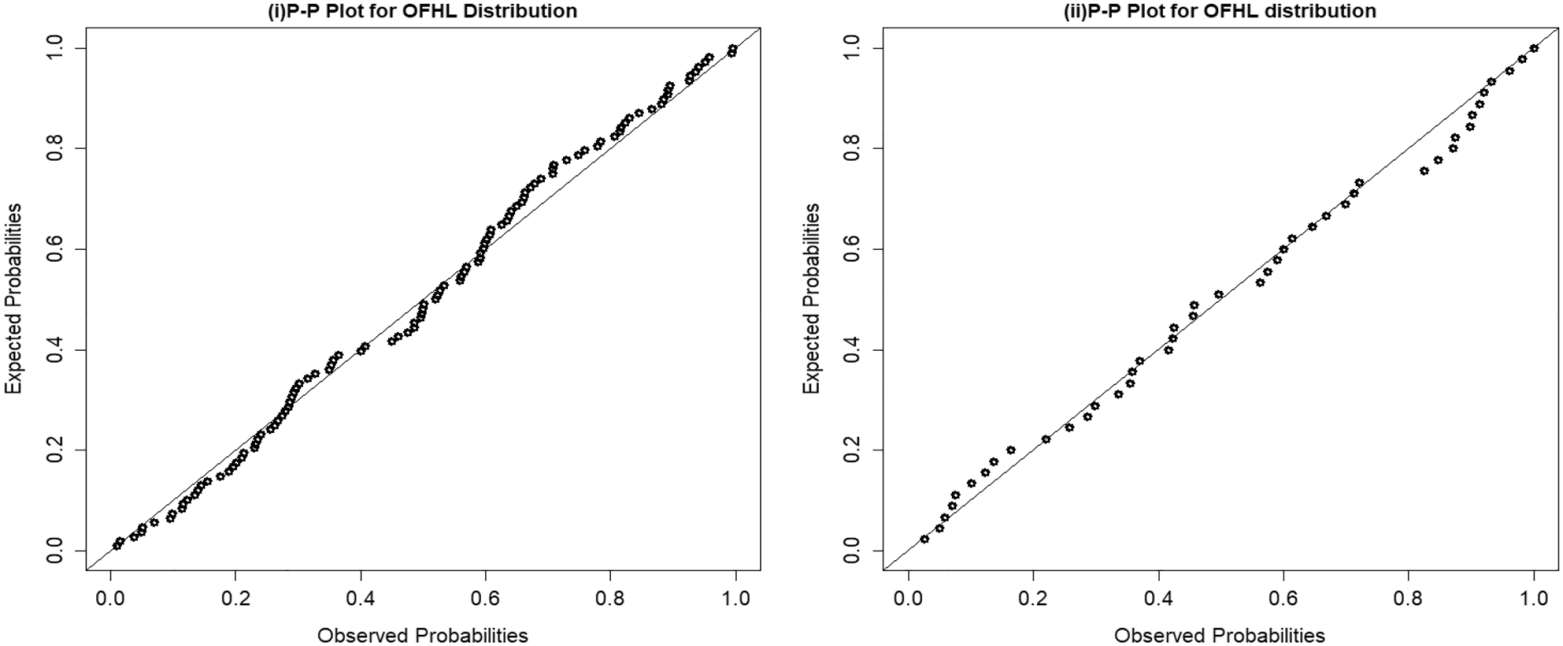
P–P plots of the OFHL for Covid-19 and Survival time datasets.

**Figure 7:**
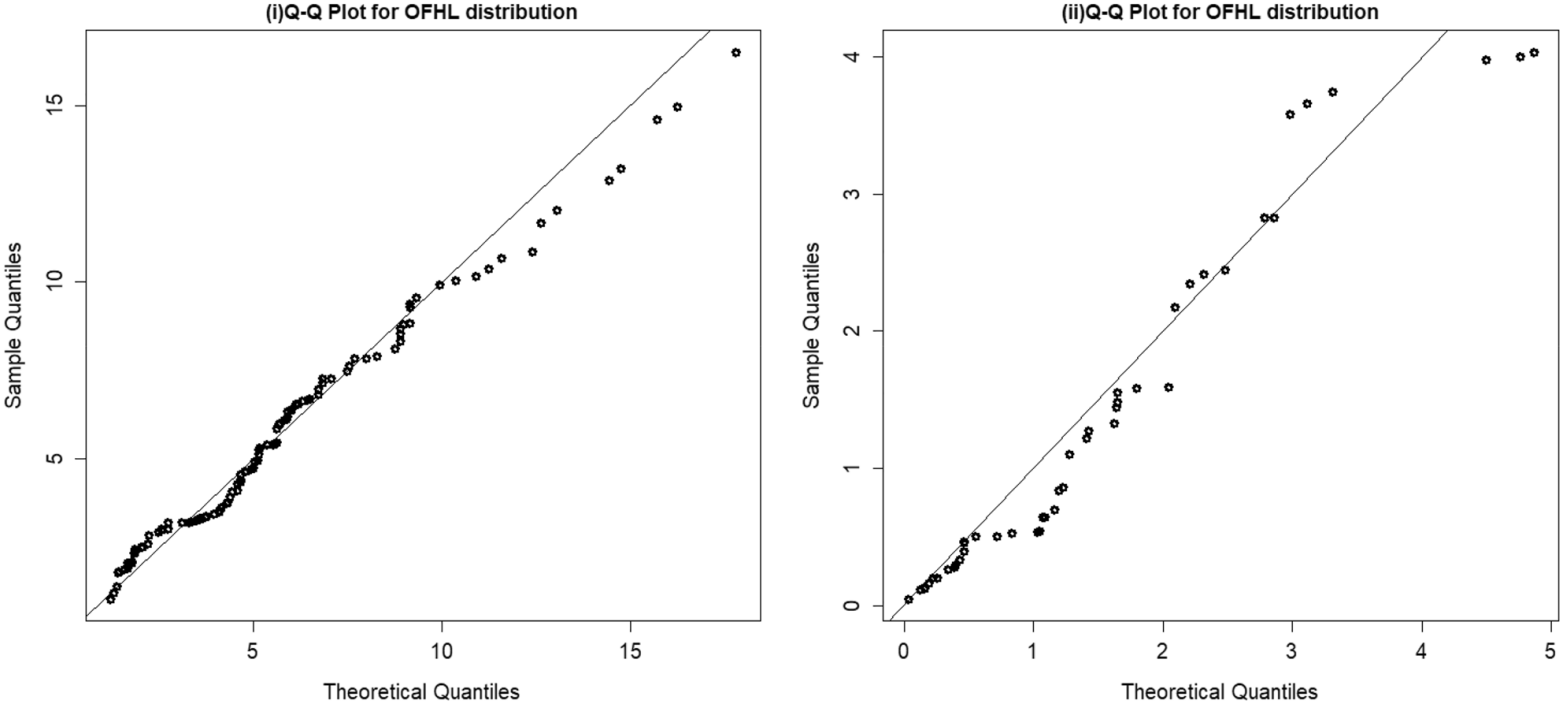
Q–Q plots of the OFHL for Covid-19 and Survival time datasets.

Furthermore, we fitted the OFHL distribution by utilizing the six estimation procedures and the results are reported in Tables 7 and 8. The estimated PDF, SF, P-P and Q-Q plots of OFHL model for two datasets are contrasted in Figs. 4, 5, 6 and 7 respectively. To sum up, the OFHL model demonstrates that it is the most appropriate model for the two datasets by illustrating how it may be applied in a real-world scenario.

**Table 7:** Estimate of Parameters and GoF metrics for Covid-19 dataset using various estimation approaches.

	$\widehat{\mathrm{\alpha }}$	$\widehat{\uptheta }$	−L	A*	W*	KS	*p*-value
MLE	1.0083	0.2807	266.453	0.3623	0.0622	0.0659	0.7316
ADE	1.0020	0.2749	266.525	0.3659	0.0628	0.0557	0.8906
CVME	0.9878	0.2734	266.611	0.3593	0.0618	0.0491	0.9567
MPSE	0.9672	0.2824	266.623	0.3367	0.0585	0.0637	0.7720
OLSE	0.9737	0.2741	266.677	0.3501	0.0605	0.0475	0.9675
WLSE	0.9985	0.2753	266.524	0.3634	0.0624	0.0556	0.8912

**Table 8:** Estimate of Parameters and GoF metrics for Survival time dataset using various estimation approaches.

	$\widehat{\mathrm{\alpha }}$	$\widehat{\uptheta }$	−L	A*	W*	KS	*p*-value
MLE	0.4835	1.7273	56.8353	0.3646	0.0515	0.1005	0.7155
ADE	0.4438	1.8046	57.0714	0.4028	0.0583	0.0857	0.8673
CVME	0.4295	1.8668	57.2771	0.4248	0.0621	0.0763	0.9378
MPSE	0.4461	1.7612	57.0547	0.3938	0.0567	0.0921	0.8065
OLSE	0.4132	1.8843	57.5716	0.4419	0.0650	0.0748	0.9462
WLSE	0.4369	1.8282	57.1610	0.4121	0.0599	0.0819	0.8986

Using the TTT (Total time on test) plot recommended by Aarset^30^, the form of the hazard function of the datasets was assessed and the results demonstrate that both datasets display different shapes of hazard rate. Iftikhar et al.^31^ also employed this technique for assessing the graphical overview of hazard rate of the data. It was introduced for the two real data sets in Fig. 3.

## Final comments on the data analysis results

- From the Tables 5 and 6, we can infer that our suggested distribution performs better as compared to other competing models.
- In dataset I and dataset II, OFHL model has the highest p-value as well as smallest AD, CVM and KS distance.

## Conclusion

In the current study, we propose a novel two parameter model, namely two-parameter odd Fréchet Half-Logistic (OFHL) distribution; its mathematical features have been thoroughly described. The OFHL distribution is more adaptable for analyzing lifespan data as compared to other models. The suggested model contains a broad variety of forms, which boosts its flexibility in modeling different types of data, as inferred from the PDF and hazard rate plots. Several conventional estimation approaches, including MLE and five other methods, were used to estimate the unknown parameters of the proposed model. A simulation study with 1000 iterations was conducted to analyse and evaluate the performance of the estimation approaches and it was found that as n increases, the estimated biases, MSEs and MREs of the parameters $\alpha \; {\text{and}} \; \theta$ under the MPSE estimation approach quickly decreases, demonstrating the effectiveness of the MPSE procedure. Further, the superiority and effectiveness of the suggested model over some of its competitors was further established using real-world data analysis, which demonstrates that the underlying model fits the data more accurately than the other distributions. We anticipate that the findings from this study will be valuable for practitioners in a variety of fields.

## Data Availability

The data that supports the findings of this study are available within the article.
